# Socioeconomic Inequalities in Type 2 Diabetes: Mediation Through Status Anxiety?

**DOI:** 10.3389/ijph.2023.1606069

**Published:** 2023-10-02

**Authors:** Loes Crielaard, Ehsan Motazedi, Henrike Galenkamp, Herman G. van de Werfhorst, Naja Hulvej Rod, Mirte A. G. Kuipers, Mary Nicolaou, Karien Stronks

**Affiliations:** ^1^ Department of Public and Occupational Health, Amsterdam UMC Location University of Amsterdam, Amsterdam Public Health Research Institute, Amsterdam, Netherlands; ^2^ Department of Political and Social Sciences, European University Institute, Florence, Italy; ^3^ Department of Public Health, University of Copenhagen, Copenhagen, Denmark

**Keywords:** mediation, socioeconomic status, type 2 diabetes, status anxiety, feelings of inferiority

## Abstract

**Objectives:** While status anxiety has received attention as a potential mechanism generating health inequalities, empirical evidence is still limited. Studies have been ecological and have largely focused on mental and not physical health outcomes.

**Methods:** We conducted individual-level analyses to assess status anxiety (feelings of inferiority resulting from social comparisons) and resources (financial difficulties) as mediators of the relationship between socioeconomic status (SES) (education/occupation/employment status) and type 2 diabetes (T2D). We used cross-sectional data of 21,150 participants (aged 18–70 years) from the Amsterdam-based HELIUS study. We estimated associations using logistic regression models and estimated mediated proportions using natural effect modelling.

**Results:** Odds of status anxiety were higher among participants with a low SES [e.g., OR = 2.66 (95% CI: 2.06–3.45) for elementary versus academic occupation]. Odds of T2D were 1.49 (95% CI: 1.12–1.97) times higher among participants experiencing status anxiety. Proportion of the SES–T2D relationship mediated was 3.2% (95% CI: 1.5%–7.0%) through status anxiety and 10.9% (95% CI: 6.6%–18.0%) through financial difficulties.

**Conclusion:** Status anxiety and financial difficulties played small but consistent mediating roles. These individual-level analyses underline status anxiety’s importance and imply that status anxiety requires attention in efforts to reduce health inequalities.

## Introduction

It is widely recognized that differences in socioeconomic status (SES)—defined based on education, income, and/or occupation—are associated with inequalities in the occurrence of chronic disease. Theories to explain the social gradient in health predominantly focus on *absolute* SES differences. Such explanations, which are well-established, pose that a lower SES implies lower access to resources and therefore worse living conditions and increased chronic stress resulting from these conditions, which contribute—directly and indirectly—to risk factors, e.g., overweight, high blood pressure, and insulin resistance, for chronic disease [1, 2]. In addition, there is increased attention for explanations that look to *relative* SES differences. These propose that a lower SES may cause poor health not only because it implies lower access to resources but also because it might induce feelings of inferiority resulting from social comparisons. The main difference between the absolute and relative explanations is that the former assume that SES differences would also affect people if they were completely isolated from each other, whereas the latter rely on social comparisons [3]. The impact of social comparisons on individual wellbeing has long been studied in the social sciences, e.g., in Merton’s (1968) Reference Group Theory [4]. The hypothesized importance of explanations that rely on relative differences for the SES–health relationship is supported by empirical evidence from countries where most people can be presumed to have sufficient access to resources but where a clear social gradient in health is still observed [5].

While there are various mechanisms that could be relevant when it comes to relative explanations for the social gradient in health (as we will lay out in the discussion of our results), the most well-known mechanism through which relative SES differences are postulated to affect health is the emotional stress response resulting from social-evaluative threat [5–8], as brought to the fore in Wilkinson and Pickett’s distinguished Psychosocial Theory. It is believed that social inequality “intensifies social hierarchies” [9] and as a consequence “drives people into social comparisons” [6, 7], causing such stress responses. This type of stress is said to result from the feeling of not counting much in the eyes of others [6], “subjectively experienced inferiority,” or “the sense of inferiority” [8] for those at the losing side of the social comparison, i.e., status anxiety (SA). However, empirical evidence for SA as a mechanism generating health inequalities is limited [7, 8]. As posed in Wilkinson and Pickett’s Psychosocial Theory, societies that are more unequal are worse off on a range of different outcomes, e.g., crime, political involvement, life satisfaction, and population health [6, 8, 10, 11]. Population-level associations between inequality and health have repeatedly been observed [12, 13]. However, in the context of this theory, SA as a mechanism connecting inequality to health has been inferred rather than demonstrated. The ecological fallacy is therefore a prominent point of critique on the Psychosocial Theory [14, 15].

Like the Psychosocial Theory, studies that *have* tested SA as a mechanism have generally been of an ecological nature: they examine how SA and health are related by comparing countries that vary in inequality. This was the case for the majority of the studies that we identified that, first, incorporated a measurement of SA and, second, investigated either the relationship between inequality and SA [7–9, 16–21] or SA as a mediator of the relationship between inequality and health [22–25]. SA was found to in part explain the relationships between inequality and happiness, life satisfaction [24], and mental wellbeing [22] but not between inequality and depression [23] and an index covering life expectancy, infant mortality, obesity, teenage birth rate, homicides, and imprisonment [25]. Moreover, the previous studies that have incorporated a measurement of SA were largely focused on mental health outcomes, i.e., happiness, life satisfaction [24], mental wellbeing [22], and depression [23]. Only one of the studies explored the mediating role of SA for physical health outcomes, i.e., life expectancy, infant mortality, obesity, and teenage birth rate, as part of an index covering six outcomes [25], finding no mediation through SA. Because inequality is not the only aspect that countries vary in and because the observed population-level associations may not apply to individuals, ecological studies are insufficient to establish SA as a mechanism generating health inequalities, as they limit causal inference. To facilitate causal inference, individual-level analyses that assess how SA depends on SES and affects health are warranted.

We therefore conduct individual-level analyses to assess SA, operationalized as feelings of inferiority, as a mediator of the relationship between SES and type 2 diabetes (T2D) using data from HELIUS, an Amsterdam-based multi-ethnic cohort study [26]. To shed further light on the relative and absolute explanations for the SES–health relationship, we additionally assess financial difficulties—as an indicator of access to resources—as a mediator of the same relationship. We opt for T2D as the health outcome because this common chronic disease has a clear social patterning [27] and the impact of chronic stress on T2D risk has been found to be especially pronounced [28]. This is relevant as SA is hypothesized to affect health through the emotional stress response. This study is informed by the following hypotheses:

H1 Socioeconomic status will be negatively associated with type 2 diabetes and status anxiety, showing a social gradient.

H2 Status anxiety will be positively associated with type 2 diabetes while controlling for socioeconomic status.

H3 Both status anxiety and financial difficulties will partly mediate the relationship between socioeconomic status and type 2 diabetes.

## Methods

### Data Collection and Study Population

Healthy Life in an Urban Setting (HELIUS) is a prospective, multi-ethnic cohort study that includes adults of Dutch, Surinamese, Moroccan, Turkish, and Ghanaian ethnic origin living in Amsterdam [26]. Participants were randomly sampled, stratified by ethnic origin, through the municipal registry of Amsterdam. The response rate was 28%, and non-response analyses indicated that SES differences between participants and non-participants were minor [26]. Of the 24,789 participants at the baseline measurement (2011–2015), 22,162 supplied questionnaire data and underwent a physical examination, including the collection of biological samples. The baseline data were used for this study. Study protocols were approved by the Amsterdam Medical Center (AMC) Ethical Review Board. Written informed consent was provided by all participants.

Participants with missing data for education (0.9% missing), employment status (1.1%), SA (0.2%), financial difficulties (0.4%), and/or T2D (0.5%) were excluded. Information on occupation was missing for 15.5% of participants. Therefore, participants with missing data for occupation were only excluded from the analyses where occupation was used to indicate SES. Participants with missing data for occupation generally did report their employment status: 83.4% of those participants described their employment status as “not in the work force,” “unemployed,” or “incapacitated.” Occupation and employment status can thus be considered to give complementing information. Participants of unknown or other ethnic origin (0.2%) were also excluded. This resulted in a study population of 21,150 participants for the analyses where education or employment status was used to indicate SES, and of 18,221 for those where occupation was used.

### Socioeconomic Status

We used education, occupation, and employment status to indicate SES. Education was based on the self-reported highest education attained either in the Netherlands or the country of origin and categorized into higher vocational schooling or university (“high”), intermediate vocational schooling or intermediate/higher secondary schooling (“medium-high”), lower vocational schooling or lower secondary schooling (“medium-low”), or no schooling or elementary schooling only (“low”). Occupation was based on self-reported job title and job description and categorized into “academic,” “higher,” “intermediate,” “lower,” or “elementary,” according to the Dutch Standard Occupational Classification system for 2010. In the case that a participant was not working at the time the questionnaire was administered, they were asked to self-report their last job. Employment status was self-reported and categorized into “paid job,” “not in the work force” (retired/studying/homemaking), “unemployed” (unemployed and looking for work/social benefit recipient), or “incapacitated” (unable to work), where “incapacitated” was considered to be the lowest employment status.

### Status Anxiety

SA was measured using the statement “I often feel I’m inferior to other people” indicated on a 5-point Likert scale. This statement is a single item, out of 12, of the Neuroticism subscale of the Neo Five Factor Inventory [26] and dovetails with the definition of SA as the feeling of not counting much in the eyes of others [6] and aligns with the statements from the European Quality of Life Survey (EQLS) that have most frequently been used to measure SA in other studies [7, 8, 17, 20, 22, 24, 25]. Specifically, in that survey, the statements 1) “Some people look down on me because of my job situation or income” and 2) “I do not feel that the value of what I do is recognized by others” indicated on a 5-point Likert scale were used.

### Financial Difficulties

Financial difficulties were measured by asking “During the past year, did you have problems managing your household income?,” with possible answers “No, no problems at all,” “No problems, but I have to watch what I spend,” “Yes, some problems,” and “Yes, lots of problems.”

### Type 2 Diabetes

Participants were considered to have T2D if at least one of the following three conditions was met: 1) they reported being diagnosed with T2D by a healthcare professional, 2) they used prescribed T2D medication, and/or 3) their fasting glucose level was ≥7 mmol/L. Use of T2D medication was assessed by asking participants to bring their prescribed medication to the physical examination. The medication was subsequently coded based on the Anatomical Therapeutical Chemical classification. Fasting glucose level was determined from fasting blood samples drawn during the physical examination.

### Ethnicity, Age, and Sex

Ethnicity was based on a participant’s registered country of birth and those of their parents, as per the standard classification of Statistics Netherlands [29]. Participants were considered of Dutch origin if they and both their parents were born in the Netherlands. Participants were considered of migrant origin if they were born abroad with at least one parent born abroad (first generation) or if they were born in the Netherlands with both parents born abroad (second generation). Age and sex were also derived from the municipal registry.

### Analyses

#### Logistic Regression Models

Associations between SES–T2D, SES–SA, and SA–T2D and equivalent paths for financial difficulties were estimated using logistic regression models implemented using R function “glm.” SA and financial difficulties were dichotomized in the analyses where they were used as dependent variables. We controlled for age (continuous), sex (binary), and ethnicity (categorical) when estimating the associations between SES–T2D, SES–SA, and SES–financial difficulties. We additionally controlled for education (ordinal) when estimating the associations between SA–T2D and financial difficulties–T2D, where we observed similar findings if controlling for occupation or employment status instead of education.

#### Mediation: Natural Effect Models

We estimated mediation of the SES–T2D relationship through SA and financial difficulties using natural effect modelling (NEM), implemented using R package “medflex” [30, 31]. The NEM approach is based on the counterfactual framework [31], an alternative method to the traditional mediation approach proposed by Baron and Kenny (1986) [32].

The NEM approach decomposes the total effect of an exposure on the outcome into natural direct and natural indirect (mediated) effects. The counterfactual variables refer to the outcome, here the absence/presence of T2D, that would have been observed if the exposure, i.e., SES, and the mediator, i.e., SA or financial difficulties, were artificially set to other possible values while every other independent variable remained the same [31]. To estimate the counterfactual variables, the NEM approach extends the actual dataset to an artificial weighted dataset, based on Hong’s (2010) ratio-of-mediator-probability weighting method [33].

In the natural effect models, we used the SES indicators as continuous exposure variables (values 1–4 for education and occupation, and 1–5 for employment status), while SA and financial difficulties were dichotomized into binary mediators. Transforming the SES indicators into continuous variables means assuming that the associations between SES–T2D, SES–SA, and SES–financial difficulties are linear. We controlled for potential confounders age (continuous), sex (binary), and ethnicity (categorical).

Mediated proportions were computed based on the odds ratios estimated for the natural direct and natural indirect effects according to [34], using:

$$\frac{{OR}^{DE}\left({OR}^{IE}-1\right)}{{OR}^{DE}{OR}^{IE}-1}$$

where ${OR}^{DE}$ and ${OR}^{IE}$ refer to direct and indirect effect odds ratios, respectively. A cut-off of 10% regarding the prevalence of the outcome is suggested for use of the formula [34]. The prevalence of T2D was 10.8% for the analyses where education or employment status was used to indicate SES, and 10.1% for those where occupation was used. The 95% Wald confidence intervals for the mediated proportions were obtained using the delta method implemented in R package “car” [35].

## Results

### Characteristics of the Study Population

Prevalence of T2D, SA, and financial difficulties are shown as stratified by education, occupation, or employment status (Table 1). The mean age was 44.4 years (SD 13.1), and 58% of participants were female.

**TABLE 1:** Characteristics of the study population [The Healthy Life in an Urban Setting (HELIUS) study, Amsterdam, Netherlands, 2011–2015].

Characteristic	% of total sample (*n* = 21,150)
Age, in years [mean (SD)]	44.4 (13.1)
Female [n (%)]	12,190 (58%)
**Ethnicity**	
Dutch [n (%)]	4,496 (21%)
Surinamese [n (%)]	7,375 (35%)
Moroccan [n (%)]	3,694 (17%)
Turkish [n (%)]	3,422 (16%)
Ghanaian [n (%)]	2,163 (10%)

	n (%) of total sample	n (%) with type 2 diabetes, within each SES category	n (%) with status anxiety^a^, within each SES category	n (%) with financial difficulties^b^, within each SES category
**Education** (*n* = 21,150)				
High [n (%)]	5,754 (27%)	260 (5%)	501 (9%)	1,131 (20%)
Medium-high [n (%)]	6,187 (29%)	443 (7%)	600 (10%)	2,321 (38%)
Medium-low [n (%)]	5,543 (26%)	797 (14%)	554 (10%)	2,551 (46%)
Low [n (%)]	3,666 (17%)	782 (21%)	569 (16%)	2,071 (56%)
**Occupation** (*n* = 18,221)				
Academic [n (%)]	1,374 (8%)	40 (3%)	98 (7%)	154 (11%)
Higher [n (%)]	3,695 (20%)	190 (5%)	301 (8%)	757 (20%)
Intermediate [n (%)]	4,851 (27%)	407 (8%)	479 (10%)	1,744 (36%)
Lower [n (%)]	5,452 (30%)	691 (13%)	605 (11%)	2,555 (47%)
Elementary [n (%)]	2,849 (16%)	512 (18%)	352 (12%)	1,467 (51%)
**Employment status** (*n* = 21,150)				
Paid job [n (%)]	12,934 (61%)	917 (7%)	1,087 (8%)	3,757 (29%)
Not in the work force [n (%)]	3,646 (17%)	537 (15%)	416 (11%)	1,383 (38%)
Unemployed [n (%)]	2,978 (14%)	430 (14%)	416 (14%)	1,913 (64%)
Incapacitated [n (%)]	1,592 (8%)	398 (25%)	305 (19%)	1,021 (64%)

^a^ For status anxiety, the options “Strongly agree” and “Agree” were taken as experience of status anxiety and the options “Neither agree nor disagree,” “Disagree,” and “Strongly disagree” as no experience of status anxiety.

^b^ For financial difficulties, the answers “Yes, lots of problems” and “Yes, some problems” referred to financial difficulties, while the answers “No problems, but I have to watch what I spend” and “No, no problems at all” referred to no financial difficulties.

Of participants with a low education, 21% had T2D, compared to 5% of participants with a high education. Similarly, 18% of participants with an elementary occupation had T2D, while this was 3% for participants with an academic occupation. Of participants that reported to be incapacitated, 25% had T2D, as compared to 7% of participants that reported to have a paid job.

SA was also more prevalent among participants with a lower SES. Of participants with a low education, 16% experienced SA, versus 9% of participants with a high education. This was 12% versus 7% for occupation, and 19% versus 8% for employment status. Similarly, financial difficulties were also reported more often by participants with a lower SES.

### Associations Between Socioeconomic Status–Type 2 Diabetes, Socioeconomic Status–Status Anxiety, and Status Anxiety–Type 2 Diabetes and Equivalent Paths for Financial Difficulties

Results of the logistic regression models (Table 2) confirmed H1: SES was negatively associated with T2D and SA, showing a social gradient. Low versus high education (OR = 2.39; 95% CI: 2.01–2.84), elementary versus academic occupation (OR = 3.76; 95% CI: 2.66–5.45), and “incapacitated” versus “paid job” employment status (OR = 2.12; 95% CI: 1.84–2.45) were associated with higher odds of having T2D. Participants with a lower SES were also more likely to experience SA: 2.39 (95% CI: 2.05–2.79) times more likely when education was used to indicate SES, and respectively 2.66 (95% CI: 2.06–3.45) and 2.83 (95% CI: 2.45–3.28) times more likely when occupation and employment status were used.

**TABLE 2:** Results of logistic regression models for the associations between socioeconomic status–type 2 diabetes, socioeconomic status–status anxiety, and status anxiety–type 2 diabetes and equivalent paths for financial difficulties [The Healthy Life in an Urban Setting (HELIUS) study, Amsterdam, Netherlands, 2011–2015].

Independent variable	Dependent variable		
	Type 2 diabetes	Status anxiety	Financial difficulties
	OR (95% CI)	OR (95% CI)	OR (95% CI)
**Education** ^a^ (*n* = 21,150)			
High	Ref.	Ref.	Ref.
Medium-high	1.36 (1.15–1.61)	1.21 (1.06–1.38)	2.00 (1.83–2.18)
Medium-low	1.80 (1.54–2.11)	1.45 (1.26–1.66)	2.64 (2.41–2.89)
Low	2.39 (2.01–2.84)	2.39 (2.05–2.79)	3.30 (2.97–3.66)
**Occupation** ^a^ (*n* = 18,221)			
Academic	Ref.	Ref.	Ref.
Higher	1.42 (1.00–2.06)	1.21 (0.96–1.55)	1.78 (1.47–2.15)
Intermediate	1.98 (1.41–2.84)	1.59 (1.27–2.02)	3.27 (2.73–3.93)
Lower	2.51 (1.80–3.59)	1.94 (1.55–2.47)	4.83 (4.04–5.82)
Elementary	3.76 (2.66–5.45)	2.66 (2.06–3.45)	5.84 (4.80–7.13)
**Employment status** ^a^ (*n* = 21,150)			
Paid job	Ref.	Ref.	Ref.
Not in the work force	1.26 (1.10–1.45)	1.29 (1.13–1.45)	1.23 (1.15–1.35)
Unemployed	1.52 (1.33–1.73)	1.89 (1.67–2.14)	3.76 (3.45–4.10)
Incapacitated	2.12 (1.84–2.45)	2.83 (2.45–3.28)	3.41 (3.05–3.82)
**Status anxiety** ^b^ (*n* = 21,150)			
Strongly disagree	Ref.	—	—
Disagree	0.99 (0.88–1.11)	—	—
Neither agree nor disagree	1.18 (1.02–1.36)	—	—
Agree	1.30 (1.08–1.56)	—	—
Strongly agree	1.49 (1.12–1.97)	—	—
**Financial difficulties** ^b^ (*n* = 21,150)			
No, no problems at all	Ref.	—	—
No problems, but I have to watch what I spend	0.98 (0.86–1.13)	—	—
Yes, some problems	1.21 (1.06–1.39)	—	—
Yes, lots of problems	1.39 (1.20–1.61)	—	—

^a^ Controlled for age, sex, and ethnicity.

^b^ Controlled for age, sex, ethnicity, and education.

The results also confirmed H2: SA was positively associated with T2D while controlling for SES (presented here for education, with similar findings while controlling for occupation and employment status). Specifically, the odds of having T2D were 1.49 (95% CI: 1.12–1.97) times higher if a participant strongly agreed than if a participant strongly disagreed with the statement “I often feel I’m inferior to other people.”

As was anticipated, equivalent paths for financial difficulties showed that SES was negatively associated with financial difficulties (education: OR = 3.30; 95% CI: 2.97–3.66; occupation: OR = 5.84; 95% CI: 4.80–7.13; and employment status: OR = 3.41; 95% CI: 3.05–3.82)—showing a social gradient—and that financial difficulties, in turn, were positively associated with T2D while controlling for SES (OR = 1.39; 95% CI: 1.20–1.61).

The odds ratios showed that the relationship between the log odds of the outcome—i.e., T2D, SA, or financial difficulties—and SES, for all indicators, was approximately linear. This supports our assumption with respect to the NEM analyses that the associations between SES–T2D, SES–SA, and SES–financial difficulties could be described by linear functions.

### Status Anxiety and Financial Difficulties as Mediators of the Relationship Between Socioeconomic Status and Type 2 Diabetes

Results of the natural effects models (Table 3) confirmed H3: both SA and financial difficulties partly mediated the relationship between SES and T2D. The reported indirect effects of SES on T2D through SA indicate that SA plays a small mediating role (OR = 1.01; 95% CI: 1.00–1.01). The proportion of the total effect of SES on T2D mediated through SA was 2.7% (95% CI: 1.4%–5.2%), 2.0% (95% CI: 1.0%–4.2%), or 3.2% (95% CI: 1.5%–7.0%) depending on whether, respectively, education, occupation, or employment status was used to indicate SES. The mediated proportion through financial difficulties was 9.6% (95% CI: 6.3%–14.8%), 9.0% (95% CI: 5.6%–14.5%), or 10.9% (95% CI: 6.6%–18.0%), with, respectively, education, occupation, or employment status as the SES indicator.

**TABLE 3:** Results of natural effect models for the mediation of the relationship between socioeconomic status and type 2 diabetes through status anxiety and financial difficulties [The Healthy Life in an Urban Setting (HELIUS) study, Amsterdam, Netherlands, 2011–2015].

Exposure	Mediator					
	Status anxiety			Financial difficulties		
	Direct effect	Indirect effect	Total effect^b^	Direct effect	Indirect effect	Total effect
**Education** ^a^ (*n* = 21,150)						
OR (95% CI)	1.32 (1.26–1.40)	1.01 (1.00–1.01)	1.33 (1.27–1.41)	1.28 (1.22–1.36)	1.02 (1.01–1.03)	1.31 (1.25–1.39)
Proportion mediated (95% CI)	2.7% (1.4%–5.2%)			9.6% (6.3%–14.8%)		
**Occupation** ^a^ (*n* = 18,221)						
OR (95% CI)	1.36 (1.29–1.44)	1.01 (1.00–1.01)	1.37 (1.29–1.45)	1.33 (1.25–1.41)	1.02 (1.01–1.04)	1.36 (1.28–1.45)
Proportion mediated (95% CI)	2.0% (1.0%–4.2%)			9.0% (5.6%–14.5%)		
**Employment status** ^a^ (*n* = 18,221)						
OR (95% CI)	1.25 (1.20–1.31)	1.01 (1.00–1.01)	1.26 (1.21–1.32)	1.25 (1.19–1.31)	1.02 (1.01–1.04)	1.28 (1.22–1.34)
Proportion mediated (95% CI)	3.2% (1.5%–7.0%)			10.9% (6.6%–18.0%)		

^a^ Controlled for age, sex, and ethnicity.

^b^ These odds ratios correspond to a one-level decrease in education, occupation, or employment status. From this it can be deduced that type 2 diabetes was 2.37 times more likely for participants with low versus high education, 3.52 times more likely for participants with elementary versus academic occupation, and 2.01 times more likely for participants with “incapacitated” versus “paid job” employment status. These odds ratios correspond approximately to those that were found using logistic regression.

## Discussion

We conducted individual-level analyses to assess SA—operationalized as feelings of inferiority—as a mediator in the relationship between SES and T2D. To shed further light on the relative and absolute explanations for the SES–health relationship, we additionally assessed financial difficulties as a mediator of the same relationship. We found that the prevalence of SA was higher among participants with a lower SES. SES was negatively associated with both T2D and SA. In turn, SA was associated with higher odds of having T2D. As hypothesized, both SA and financial difficulties played small but consistent mediating roles in the SES–T2D relationship. The mediated proportion through SA (2%–3%) was smaller than through financial difficulties (9%–11%).

These results should be seen in light of a number of limitations. First, all analyses were cross-sectional, where SES, SA, financial difficulties, and T2D were measured simultaneously. This prohibits establishing causality. Reverse causality is possible, e.g., those living with T2D may feel inferior due to their disease or its consequences (e.g., unemployment), and low SES may result from disease. Second, although a natural effect model is considered a non-parametric structural equation model, it still relies on the assumption that there are “no unmeasured confounders for the exposure-outcome, exposure-mediator, or mediator-outcome relations” [31]. This is particularly difficult to ensure for the mediator-outcome relations, as there are factors, e.g., underlying mental health problems, that may affect both SA and T2D risk. Mental health problems may also be possible consequences of SES, making them “exposure dependent confounders,” which are complicated to adjust for. Third, SA was measured by a single item, out of 12, of the Neuroticism subscale of the Neo Five Factor Inventory. While it may not be optimal to use a single item to operationalize SA, it is not unprecedented [8, 22]. Fourth, building on that, this measurement was not designed to capture SA. It is possible that participants perceived the statement “I often feel I’m inferior to other people” as normative, which could cause them to refrain from agreeing with the statement. Nevertheless, we did find that SA was prevalent in the entire population. In addition, while the statement does include a social subordination element, i.e., “I often feel I’m inferior *to other people*,” it does not specify that this is caused by relative SES differences (e.g., “More educated people look down on people like me”). The frequently used EQLS measurement does particularize a SES element—i.e., in item (1) “Some people look down on me *because of my job situation or income*.” The observed social gradient in SA as it was operationalized in this study does build confidence in the use of the measurement in the context of relative SES differences. Furthermore, it could conversely be argued that leaving the causes of the feelings of inferiority undefined functions as an advantage. Specifically, as considered by Delhey et al. (2017) in their discussion of the EQLS measurement, if the SES elements are particularized, “the phrasing may favor job and income as potential reasons for status anxiety” [7], above other indicators, e.g., education and employment status. Another strength of the statement used is that it reflects possible feelings attached to social position—in contrast to, e.g., analyses that use subjective social status (SSS) as an indicator for how people perceive themselves in relation to others or analyses that only rely on item (1) from the EQLS [8, 22]. That is, a low SSS does need to be accompanied by feelings of inferiority. To address possible measurement errors and account for any potential noisiness of the SA measurement, the existence of which we cannot ascertain in this study, we opted for dichotomizing SA—as well as financial difficulties—in order to function as a more robust indicator. To ascertain that this decision did not distort the results, we repeated the NEM analyses employing SA and financial difficulties as un-dichotomized, continuous mediators and found that this yielded similar relative results, i.e., a mediated proportion of 3%–5% through SA and of 11%–12% through financial difficulties (see Supplementary Table S1 in the Additional material for the results with the un-dichotomized mediators).

Much like previous ecological studies, these individual-level analyses imply that relative SES differences play a role in health inequalities. The notion that resource-based explanations are only part of the story is supported by our finding that, in this study, about 10% of the SES–T2D relationship could be ascribed to financial difficulties. While the proportion of the SES–T2D relationship mediated by SA was smaller than the proportion mediated by financial difficulties, the consistent association observed across different SES indicators indicates a robustness of this relationship. Furthermore, the proportion of the SES–T2D relationship mediated by SA may seem small but is of the same order of magnitude as the proportion attributable to other, widely accepted mediators, e.g., smoking—for which the reported proportion mediated ranges from 1% to 13% [36–38].

This study substantiates the theory that social comparisons are connected to individual wellbeing: feeling inferior to other people, resulting from social comparisons, was associated with T2D. Although these individual-level analyses support SA as a mechanism generating health inequalities, complementary analyses are required to further our understanding of the relationship between inequality, SA, and individual wellbeing. Arguably, a variety of analyses should be used in combination to appreciate how different constructs of inequality affect individual wellbeing and to eventually understand what we could do to address this.

First, while our analyses provide insight into how people *perceive* inequality, multilevel analyses using, e.g., the Gini coefficient could be used to ascertain whether more inequality at the societal level leads to more SA among individuals [2, 39].

Second, while our analyses link SES to SA and T2D, research has shown that, when it comes to health, results in relation to ‘objective’ SES do not always correspond to those in relation to SSS [1]. Analyses that incorporate SSS can provide additional information about the relationship between social position and SA. In addition, the concept of SSS relates to debates about the importance of the terminology used to refer to different types of education and occupation in that it may carry a value judgement. For example, in the Netherlands, it has been argued that instead of “low” and “high” education, the terms “practically trained” and “theoretically trained” should be used [40], consistent with the idea that they differ but should be equally valued by society.

Third, while the majority of identified studies operationalized SA as feelings of inferiority [7, 8, 17, 20, 22, 24, 25], other aspects of SA could be put at the forefront, which may lead to additional insights. Aspects identified as important may include status seeking [9, 18, 19, 41], desire for wealth and status [21], financial satisfaction and perceived relative income [42], economic worries [43], and perceived importance of income comparisons [23]. That other aspects of SA may lead to additional insights is reflected in the diversity in findings from various studies. For instance, Paskov et al. (2017) found that status seeking was more pronounced among those with a high social position and less prevalent among the unemployed [9], while Delhey, Schneickert, and Steckermeier (2017) and Layte and Whelan (2014) found that feelings of inferiority occurred more often among those with a low income [7, 8] and those excluded from the labor market [7]. Moreover, SA has also been defined as “a worry that we are currently occupying too modest a rung [on the social ladder] *or are about to fall to a lower one*” [44], implying the importance of the fear of losing one’s social position as an aspect of SA. Day and Fiske (2016) have introduced, as recently published by Melita et al. (2020), a designated Status Anxiety Scale consisting of five items covering different aspects of SA, e.g., “I worry that my social status will not change” and “I sometimes worry that I might become lower in social standing” [45, 46]. This has been employed in recent studies showing that SA uniquely explained job satisfaction [47], that the relationship between perceived economic inequality and SA is mediated by perceived competitiveness [16], and, through experiments, that the relationship between perceived economic inequality and consumption is mediated by status seeking [48].

Fourth, while the Psychosocial Theory has focused attention on SA, SA is arguably not the only possible cause of the social gradient in health that depends on the way society is organized. For example, the belief that we live in a meritocracy, a society in which people acquire their status based on their talents and efforts, could affect how people feel about their own social position. Other relative explanations for the social gradient in health should also be considered. For example, the Fundamental Cause Theory of health disparities includes power and prestige as assets that are primarily held by those with a high SES [49]. Here, power and prestige are presumably only favorable if they exceed the power and prestige of others. Apart from SA, growing relative SES differences could also breed feelings of unfairness. Such feelings may become increasingly influential in light of the “disproportionate share of global wealth growth” that has been attained by multimillionaires over the last 3 decades and especially during the COVID-19 pandemic [50]. In this regard, Bosma et al. (2012) found, while an effect of SES on health *via* perceived unfairness could not be detected, that perceived unfairness was more commonly experienced by people with a lower SES and that perceived unfairness at baseline, in turn, was related to worse mental as well as physical functioning 7 years later [51].

### Conclusion

SA was prevalent in the entire population but was more prevalent among participants with a low SES. Both SA and financial difficulties played small but consistent mediating roles in the SES–T2D relationship. Although the analyses were cross-sectional, the results indicated that a lower SES may cause poor health not only because it implies lower access to resources but also because it might induce feelings of inferiority resulting from social comparisons. These individual-level analyses underline the importance of SA and imply that SA and potentially other negative consequences of social comparisons require attention in efforts to reduce health inequalities.
