# Supplementary material for: Socioeconomic Inequalities in Type 2 Diabetes: Mediation Through Status Anxiety?
Source: Int J Public Health. 2023 Oct 2;68:1606069. doi: 10.3389/ijph.2023.1606069 (PMC10577225; doi:10.3389/ijph.2023.1606069)
Supplement: Supplementary file 1 [file Table1.docx]

**TABLE A.1 |** Results of natural effect models for the mediation of the relationship between socioeconomic status and type 2 diabetes through status anxiety and financial difficulties with un-dichotomized mediators (as opposed to dichotomized mediators in the main manuscript) (The Healthy Life in an Urban Setting (HELIUS) study, Amsterdam, Netherlands, 2011-2015).

| Exposure | Mediator | | | | | |
| --- | --- | --- | --- | --- | --- | --- |
|  | **Status anxiety** | | | **Financial difficulties** | | |
|  | **Direct effect** | **Indirect effect** | **Total effect^b^** | **Direct effect** | **Indirect effect** | **Total effect** |
| **Education^a^ (n = 21,150)** | | | | | | |
| OR (95% CI) | 1.32 (1.25-1.39) | 1.01 (1.00-1.01) | 1.33 (1.26-1.40) | 1.27 (1.20-1.35) | 1.03 (1.02-1.04) | 1.31 (1.24-1.38) |
| Proportion mediated (95%CI) | 3.8% (2.2%-6.7%) | | | 12.3% (8.1%-18.6%) | | |
| **Occupation^a^ (n = 18,221)** | | | | | | |
| OR (95%CI) | 1.36 (1.28-1.44) | 1.01 (1.00-1.01) | 1.37 (1.29-1.45) | 1.32 (1.23-1.40) | 1.03 (1.02-1.04) | 1.35 (1.27-1.44) |
| Proportion mediated (95%CI) | 3.1% (1.6%-5.9%) | | | 10.8% (6.5%-17.9%) | | |
| **Employment status^a^ (n = 18,221)** | | | | | | |
| OR (95% CI) | 1.25 (1.20-1.31) | 1.01 (1.00-1.02) | 1.26 (1.21-1.32) | 1.25 (1.19-1.32) | 1.03 (1.01-1.04) | 1.28 (1.22-1.35) |
| Proportion mediated (95%CI) | 5.3% (2.9%-9.5%) | | | 11.7% (6.3%-21.6%) | | |

^a^Controlled for age, sex, and ethnicity.
^b^These odds ratios correspond to a one-level decrease in education, occupation, or employment status. From this it can be deduced that type 2 diabetes was 2.37 times more likely for participants with low versus high education, 3.52 times more likely for participants with elementary versus academic occupation, and 2.01 times more likely for participants with “incapacitated” versus “paid job” employment status. These odds ratios correspond approximately to those that were found using logistic regression.
